# Perineal Accessory Scrotum with Congenital Lipoma: A Rare Case Report

**DOI:** 10.1155/2012/757120

**Published:** 2012-08-05

**Authors:** Souvik Chatterjee, Vishal Gajbhiye, Sasanka Nath, Dipak Ghosh, Sarbani Chattopadhyay, Sukanta Kumar Das

**Affiliations:** ^1^Department of General Surgery, Medical College and Hospital, Kolkata, 88 College Street, Kolkata-700073, West Bengal, India; ^2^Department of Pathology, Medical College and Hospital, Kolkata, 88 College Street, Kolkata-700073, West Bengal, India

## Abstract

A case of accessory scrotum in a 1-year-old boy is reported because of its rarity. A boy presented with a tumor mass attached with scrotum-like skin on its tip in the right side of perineum between the scrotum and anus. Both testes had descended into the scrotum. There was no other urological anomaly. Histological findings of the tumor indicated perineal lipoma, and the scrotum-like portion accessory scrotum. An overview of sequences during the normal development of male external genitalia has been provided and the deranged mechanism resulting in this anomaly has been reviewed with hypothesis regarding etiology of accessory scrotum.

## 1. Introduction

 Accessory scrotum is considered the rarest of all congenital scrotal abnormalities [1]. Inaccessory scrotum, in addition to a normally developed scrotum, ectopic scrotal tissue is present either in the perineum or elsewhere, without the presence of testis within it [2, 3]. Accessory scrotum has been observed in isolation or in conjunction with other anorectal/urogenital abnormalities [1]. An interesting case of accessory scrotum in the perineum with a perineal lipoma is reported with the relevant embryological basis for this condition, and hypotheses for the development of an accessory scrotum have been discussed.

## 2. Case Report

**Figure 1:**
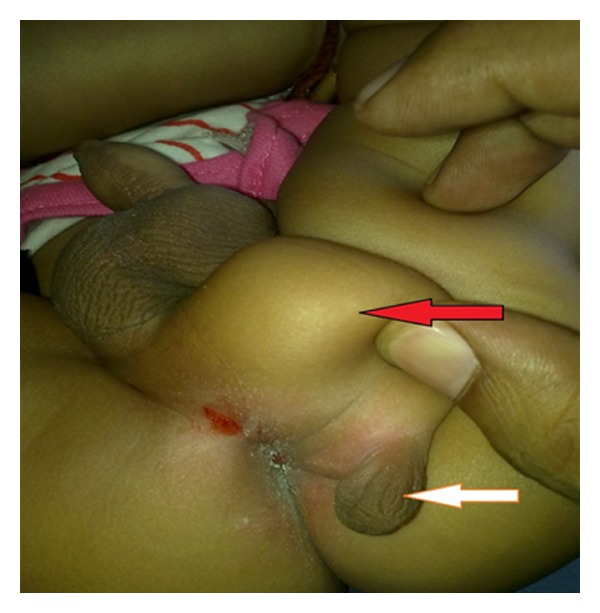
Red arrow shows intervening lipoma and white arrow shows accessory scrotum.

A 1-year-old boy presented with a mass localized to the right inferior aspect of genitalia in the perineal region, another small swelling was attached to the undersurface of the mass (Figure 1). Both mass and swelling were present since birth. The mother's health throughout pregnancy had been uneventful, and there had been no recognized exposures to any teratogenic agents.

The penis and the primary scrotum were completely normal, were contained testes within the scrotum. The mass just below the scrotum was soft in consistency and freely mobile. The swelling attached below the mass was soft, rugose, and contained no discernable testis-like structures. There was no other urological anomaly.

**Figure 2:**
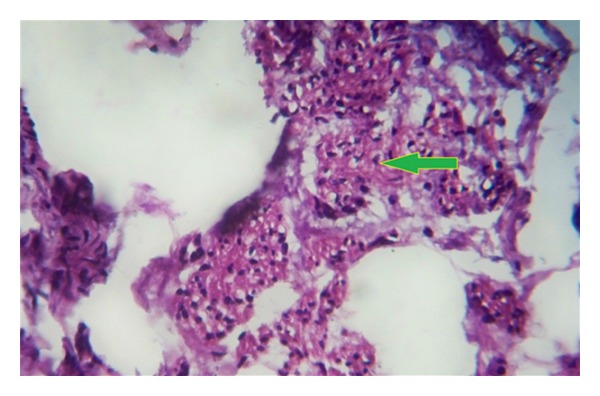
Arrow shows rudimentary dartos muscles.

The mass and swelling were surgically removed, and postoperative recovery was uneventful. The histological examination revealed the mass as being lipoma, and the other swelling had rugose epidermis with hair follicles and rudimentary dartos fibers, so histopathological examination confirmed the diagnosis of accessory scrotum (Figure 2).

## 3. Discussion

Accessory scrotum is an extremely rare abnormality. Congenital scrotal anomalies are conventionally classified into four types: bifid scrotum, penoscrotal transposition, ectopic scrotum, and accessory scrotum. Bifid scrotum is a partial or complete separation of otherwise normally positioned hemiscrotum in patients with severe hypospadias or chordee. In cases of penoscrotal transposition, part or whole of the scrotum is located superior to the penile shaft. Ectopic scrotum is ectopic positioning of the scrotum which is usually unilateral, with the ectopic tissue usually suprainguinal, but in some cases infrainguinal (femoral) or on the thigh. The ipsilateral testis is usually present within the ectopic hemiscrotum. Inaccessory scrotum, in addition to a normally developed scrotum, ectopic scrotal tissue is present either in the perineum or elsewhere, without the presence of testis within it. About 30 cases have been reported in the literature either being solitary or in association with other urogenital or nonurogenital abnormalities [4]. In the absence of a perineal lipoma, accessory perineal scrota are usually associated with other anomalies, including hypospadias, diphallia, defects of scrotal position, anorectal anomalies, and the VACTERL (vertebral, anal, cardiac, tracheoesophageal, renal, and limb anomalies) association [5].

 Male external genital development depends on the conversion of testosterone to the more active dihydrotestosterone and its subsequent action via tissue receptors. The genital tubercle enlarges into the penis. The scrotum forms from the labioscrotal swellings at fourth week of gestation. The labioscrotal swellings migrate inferomedially and merge at 12 weeks of gestation to form the scrotum, with the line of fusion being the scrotal raphe. 

The etiology for an accessory scrotum is not known. There are two common hypotheses for the development of an accessory scrotum. Lamm and Kaplan postulated that one labioscrotal swelling may embryologically divide into two portions with the inferior portion migrating incompletely to form an accessory scrotum [6]. According to Sule et al., the accessory labioscrotal fold usually develops due to intervening mesenchymal tissue disrupting the continuity of developing labioscrotal swelling [7].

It appears probable that the condition presented here has been a result of intervening mesenchymal tissue (lipoma) disrupted the continuity of developing labioscrotal swelling. Absence of multiple organ malformations suggests that the causative factor for this anomaly did not have adverse effects on other organ systems getting differentiated simultaneously, like the musculoskeletal system, spine and central nervous system, even though all these structures get differentiated during the same gestational period.
